# Depression Symptoms, Perceived Stress, and Loneliness During the COVID-19 Pandemic Among Diverse US Racial-Ethnic Groups

**DOI:** 10.1089/heq.2022.0178

**Published:** 2023-06-15

**Authors:** Anna María Nápoles, Anita L. Stewart, Paula D. Strassle, Alia Alhomsi, Stephanie Quintero, Stephanie Ponce, Miciah Wilkerson, Jackie Bonilla

**Affiliations:** ^1^ Division of Intramural Research, National Institute on Minority Health and Health Disparities, National Institutes of Health, Bethesda, Maryland, USA.; ^2^ Center for Aging in Diverse Communities, Institute for Health and Aging, University of California San Francisco, San Francisco, California, USA.

**Keywords:** COVID-19, psychological distress, race-ethnicity, national survey, black/African American, Hispanic/Latino, Asian, American Indian/Alaska Native, Native Hawaiian/Pacific Islander

## Abstract

**Introduction:**

Studies have reported increases in psychological distress during the COVID-19 pandemic. This study aimed to estimate associations between race-ethnicity and psychological distress during the COVID-19 pandemic among nationally representative samples of all major racial-ethnic groups in the United States.

**Methods:**

We conducted a nationally representative cross-sectional survey between December 2020 and February 2021 of Asian, black/African American, Latino (English and Spanish speaking), American Indian/Alaska Native, Native Hawaiian/Pacific Islander, white, and multiracial adults (*n*=5500). Distress measures included: anxiety-depression (Patient Health Questionnaire-4 [PHQ-4]), stress (modified Perceived Stress Scale), and loneliness-isolation (frequency felt lonely and isolated). Multinomial logistic regression models estimated associations between race-ethnicity and psychological distress, adjusting for demographic and health characteristics.

**Results:**

Overall, 23.7% reported moderate/severe anxiety-depression symptoms, 34.3% reported moderate/severe stress, and 21.3% reported feeling lonely-isolated fairly/very often. Compared with white adults and adjusting for covariates, the prevalence of moderate/severe anxiety-depression was significantly lower among Asian (adjusted odds ratio [aOR]=0.44, 95% confidence interval [CI]=0.34–0.58), black (aOR=0.49, 95% CI=0.38–0.63), English-speaking Latino (aOR=0.62, 95% CI=0.45–0.85), Spanish-speaking Latino (aOR=0.31, 95% CI=0.22–0.44), and Native Hawaiian/Pacific Islander (aOR=0.66, 95% CI=0.49–0.90) adults. Similar trends were seen for moderate/severe stress and feeling lonely-isolated fairly/very often. Worse distress profiles of American Indian/Alaska Native and multiracial adults were attenuated after adjustment.

**Conclusions:**

Minoritized groups tended to have less distress than white adults. Collective experiences of cumulative disadvantage could engender shared resiliency/normalization among these groups.

## Introduction

The COVID-19 pandemic has disrupted lives across many domains, including relationships, work, finances, housing, and health care, leading the American Psychological Association to declare a national mental health crisis in the United States.^1,2^ Mitigation measures of social distancing and stay-at-home orders have contributed to disruptions and social isolation. Studies have reported increases in anxiety, depression, stress, and loneliness, with nearly 8 in 10 Americans indicating that the pandemic was a significant source of stress.^1,3,4^

Structural racism and social inequities experienced by racialized US ethnic groups could contribute to higher levels of psychological distress during the pandemic among these groups compared with white adults. The mechanisms of psychological distress are multifactorial, with some being shared across racial-ethnic groups and others being specific to certain groups, generally due to structural and historical inequities experienced by these groups. Importantly, some racial-ethnic minority groups demonstrate protective factors that can serve as buffers of traumatic events, although these factors are understudied relative to factors that increase risk of distress. Several studies during the pandemic found a higher prevalence of psychological distress among Latino persons than white persons.^3,5–11^

One study among adults ages 55+ found higher levels of psychological distress and higher prevalence of COVID-19 stressors (i.e., income loss, housing insecurity, and food insecurity) among black, Asian, and Latino individuals than among white individuals.^10^ However, some groups have not been included (e.g., American Indian/Alaska Native peoples) and distinctions between Spanish- and English-speaking Latino individuals have not been made.

Racial-ethnic minority groups in the United States tend to have lower levels of income, education, and wealth on average, compared with the white populations, which in turn could make them more vulnerable to economic stressors and job disruptions imposed by the pandemic.^11^ Overall, black and Latino adults report lower household incomes and less income-producing wealth than white adults, with large racial-ethnic income gaps persisting for more than 20 years (1988–2009).^12^ According to the Conservation of Resources theory, persons are driven to protect valued survival resources, and when traumatic events occur such as natural disasters or pandemics that cause resource losses, the mental health consequences are expected to be disproportionately higher among persons of color due to long-term structural inequities.^10,13^

During the COVID-19 pandemic, racial-ethnic minority adults experienced greater risks of COVID-19 infection, hospitalization, and mortality, most likely due to poverty, poor and crowded housing conditions, financial stress, and speaking a language other than the dominant national language.^14^ Consistent with the Conservation of Resources theory, early reports during the COVID-19 pandemic indicate that being from a low-income household, greater exposure to life stressors due to the pandemic (e.g., job loss, death of someone close to you due to COVID-19), and being of black or Latino race-ethnicity were associated with a greater risk of depression symptoms.^4,8^

We conducted a nationally representative survey among American Indian/Alaska Native, Asian, black/African American, Latino (Spanish and English speaking), Native Hawaiian/Pacific Islander, white, and multiracial adults during a dramatic peak of the pandemic (December 2020–February 2021) to examine its impact across diverse racial-ethnic groups. The aims of this analysis were to: (1) estimate the prevalence of anxiety-depression symptoms, perceived stress, and loneliness overall; (2) examine whether the prevalence varies by racial-ethnic group and language (for Latino groups); and (3) describe the impact of the COVID-19 pandemic on their lived experiences of psychological distress based on responses to an open-ended question asking about changes in their lives due to the pandemic.

## Methods

### COVID-19's Unequal Racial Burden study

The COVID-19's Unequal Racial Burden (CURB) study was a nationally representative, cross-sectional online survey to determine the impact of the COVID-19 pandemic among US adults from diverse racial-ethnic groups, sampling 1000 each of Asian, black/African American, Latino (500 Spanish speaking), and white adults (≥18 years old), and 500 each of American Indian/Alaska Native, Native Hawaiian/Pacific Islander, and multiracial adults. The CURB survey was conducted by YouGov, a consumer research firm, between December 8, 2020, and February 17, 2021. YouGov uses a proprietary, opt-in survey panel (members agree to be contacted for surveys) that comprised more than 1.8 million US residents. Details about the panel and sampling methods are described elsewhere.^15,16^ YouGov has received Federalwide Assurance by the Department of Health and Human Services (FWA00010960) and adheres to 45 code of federal regulations part 46 regulations.

The National Institutes of Health Office of IRB Operations issued a determination on 08/14/2020 (IRB no. 000166) that this study does not qualify as human subjects research because YouGov provided deidentified data to the researchers. For the CURB survey, before they could proceed with the survey, potential participants were sent an email invitation that presented required informed consent elements and asked them to click on a “yes” response if they agreed to participate.

### Dependent variables

We measured three aspects of psychological distress: anxiety-depression, perceived stress, and loneliness-isolation. Anxiety-depression symptoms were measured using the Patient Health Questionnaire-4 (PHQ-4),^17^ which asks how often in the past 2 weeks they were bothered by feeling nervous/anxious, not able to control worrying, little interest or pleasure, and feeling depressed or hopeless (response choices: 0=not at all, 1=several days, 2=more than half the days, 3=nearly every day). A summed score ranges from 0 to 12 with higher scores indicating more symptoms; scores are categorized into normal (0–2), mild (3–5), moderate (6–8), and severe (9–12).^17^ Because 33% reported a score of 0, we split the normal category into 0 (none) and 1–2 (very mild). For modeling purposes, we further collapsed these to none (reference), very mild/mild, and moderate/severe.

The PHQ-4 has demonstrated excellent psychometric properties in primary care patients^17^ and the general population,^18^ and has been validated in Spanish and English languages among Latino adults. Evidence exists of the validity and reliability of the longer PHQ-9 (which contains the PHQ-4) for use among Chinese American, Latino, and African American adults using either English or translated versions.^20^

The perceived stress measure consisted of the six negatively worded questions from the Perceived Stress Scale-10 (PSS-10).^21^ All three versions of the PSS (14-, 10-, and 4-item versions) have been used widely in a large variety of populations.^22^ A two-factor solution (negative and positive subscales) is consistently found to be optimal.^23^ We used a Spanish translation previously validated in our own research with Spanish-speaking Latinas.^24^ The PSS has been validated among Koreans (in both English and Korean)^25^ and African Americans.^26^

PSS items measure the extent in the past month they felt unable to control things, stressed/nervous, could not cope, upset by unexpected event, angered by things they are unable to control, and could not overcome difficulties (response choices: 1=never, 2=almost never, 3=sometimes, 4=fairly often, 5=very often). Items are averaged for a score ranging from 1 to 5; higher scores indicate more stress. We categorized stress as follows: low (1–<2), mild (2–<3), moderate (3–<4), and severe (≥4). For modeling purposes, we further collapsed these to low (reference), mild, and moderate/severe stress.

For loneliness-isolation, we used a new single-item measure asking how often in the past month they felt lonely and isolated (response choices: 1=never, 2=almost never, 3=sometimes, 4=fairly often, 5=very often). For modeling purposes, we collapsed responses to never (reference [1]), almost never/sometimes (2–3), and fairly/very often (4–5).

#### Open-ended question on impact of pandemic

Respondents were asked: “Is there anything else about your life that has changed due to the COVID-19 pandemic that we did not ask?” allowing them to type in an answer.

### Independent variable

Self-identified race-ethnicity was measured with “Which one of the following would you say best represents your race-ethnicity?” Response options were Latino/a/x or Hispanic, American Indian or Alaska Native, Asian, black or African American, Native Hawaiian or Pacific Islander, white, or multiracial. Those selecting Asian, Latino, or Native Hawaiian/Pacific Islander were also asked to identify one relevant national origin group. National origin group options for Asian respondents were Asian Indian, Chinese, Filipino, Japanese, Korean, Vietnamese, and other Asian. Due to small sample sizes, Korean and Vietnamese participants were combined with the other Asian participants.

For Latino participants, response options included Mexican/Mexican American/Chicano, Puerto Rican, Cuban/Cuban American, Dominican, Central American, South American, and other Hispanic, Latino, or Spanish origin. Spanish- and English-speaking status among Latino participants was based on preferred survey language. Native Hawaiian/Pacific Islander participants could select Native Hawaiian, Guamanian or Chamorro, Samoan, and other Pacific Islander. Guamanian or Chamorro, Samoan, and other Pacific Islander participants were combined for analyses.

### Other covariates

Demographic variables included: age (18–29, 30–39, 40–49, 50–59, 60–69, and ≥70), gender (man, woman, transgender, or nonbinary (nonbinary, gender fluid, gender queer, other, and no gender), marital status (married/domestic partnership vs. unmarried), immigration status (US-born citizen, foreign-born citizen or legal resident, undocumented), and English proficiency (limited, not limited; limited=speaks English “not at all,” “poorly,” or “fairly well”). Socioeconomic status measures were as follows: educational attainment (<high school, high school/general educational development, some college/vocational school, college graduate or more), family annual income (<$20,000, $20,000–$59,999, $60,000–$99,999, ≥$100,000), and health insurance (private insurance, public insurance only, uninsured).

Self-reported physical health was dichotomized as fair/poor versus excellent/very good/good. Comorbidities (any vs. none) were captured by asking whether they had ever been told by a medical doctor that they had a list of conditions (selected due to their conferring increased risk of serious COVID-19 as per the Center for Disease Control and Prevention, December 2020) in the past year: chronic obstructive pulmonary disease, chronic kidney disease/on dialysis, type 2 diabetes, heart conditions, immunocompromised state from solid organ transplant, obesity, and sickle-cell anemia.

### Statistical analyses

Descriptive statistics were used to characterize the sample and examine differences in psychological distress (anxiety-depression symptoms, perceived stress, and loneliness-isolation) by racial-ethnic group and demographics. Cochran–Armitage trend tests were used to compare psychological distress severity between each racial-ethnic minority group and white adults. A *p*-value <0.05 was considered statistically significant.

Multinomial logistic regression models, adjusting for age, gender, education, and self-reported physical health, estimated the odds of reporting very mild/mild or moderate/severe anxiety-depression symptoms (reference=none) for each of the diverse racial-ethnic groups, compared with white adults. Perceived stress (mild or moderate/severe [reference=low]) and loneliness (almost never/sometimes or fairly often/very often [reference=never]) were modeled similarly.

For the open-ended question, verbatim responses were coded independently using a grounded theory approach in two steps by at least two coders.^27^ In the first step, coders extracted the subset of responses related to mental health using open coding methods (breaking the data into discrete parts and creating codes to label them). Then, applying axial coding methods (codes were organized into categories/themes), the subset of responses related to mental health was coded for salient themes, definitions of themes, and illustrative quotes. Consensus was reached through iterative meetings.

## Results

### Anxiety-depression symptoms

In the total sample (*n*=5500), about a fourth (23.7%, *n*=1302) reported moderate/severe levels of anxiety-depression symptoms (Table 1). Compared with white adults (moderate/severe=25.6%), American Indian/Alaska Native (32.1%) and multiracial adults (29.0%) were more likely, and Asian (18.1%), black (22.9%), and Spanish-speaking Latino (15.2%) adults were less likely to report moderate/severe anxiety-depression (all *p*<0.05). Spanish-speaking Latino adults were less likely to report anxiety-depression symptoms than English-speaking Latino adults, *p*=0.0007. Among the Latino subpopulations, Puerto Rican adults had the highest prevalence of moderate/severe anxiety-depression (31.2% vs. <20% for all the rest).

**Table 1.** Participant Characteristics, Weighted to Be Nationally Representative Within Racial-Ethnic Groups, Stratified by Anxiety-Depression Symptom Severity

	Anxiety-depression*^a^*				
	*Overall*	*None*	*Very mild/mild*	*Moderate/severe*	****p*** ^b^*
Total, *n*	5489	1824 (33.2)	2363 (43.0)	1302 (23.7)	—
Race-ethnicity, *n* (%)					
American Indian/Alaska Native	500 (9.1)	129 (26.0)	209 (41.9)	160 (32.1)	0.001
Asian	1000 (18.2)	366 (36.6)	452 (45.3)	181 (18.1)	0.001
Asian Indian	175 (3.2)	47 (27.0)	90 (51.8)	37 (21.2)	—
Chinese	274 (5.0)	108 (39.3)	129 (47.2)	37 (13.5)	—
Filipino	161 (2.9)	64 (39.7)	60 (37.2)	37 (23.1)	—
Japanese	132 (2.4)	60 (45.6)	56 (42.1)	16 (12.3)	—
Other Asian	258 (4.7)	87 (33.8)	117 (45.5)	53 (20.7)	—
Black/African American	1000 (18.2)	388 (38.9)	381 (38.2)	228 (22.9)	0.01
Latino	1000 (18.2)	350 (35.0)	454 (45.5)	195 (19.5)	0.02
*English speaking*	496 (49.6)	157 (31.6)	221 (44.6)	118 (23.8)	0.95
*Spanish speaking*	504 (50.4)	193 (38.4)	233 (46.4)	76 (15.2)	<0.0001
Mexican/Mexican American/Chicano	528 (9.6)	179 (34.1)	254 (48.1)	94 (17.8)	—
Puerto Rican	115 (2.1)	35 (30.6)	44 (38.2)	36 (31.2)	—
Cuban/Dominican Republic	85 (1.5)	32 (38.2)	37 (43.9)	15 (17.9)	—
Central American	80 (1.4)	27 (33.3)	38 (48.3)	15 (18.4)	—
South American	99 (1.8)	38 (38.3)	44 (44.6)	17 (17.0)	—
Other Hispanic/Latino	94 (1.7)	38 (40.8)	37 (39.5)	19 (19.7)	—
Native Hawaiian/Pacific Islander	500 (9.1)	165 (33.1)	196 (39.3)	138 (27.6)	0.63
Native Hawaiian	274 (5.0)	93 (34.0)	107 (39.2)	73 (26.8)	—
Pacific Islanders	226 (4.1)	72 (32.0)	89 (39.3)	65 (28.7)	—
White	1000 (18.2)	331 (33.1)	411 (41.2)	256 (25.6)	(ref)
Multiracial	500 (9.1)	95 (19.0)	259 (52.0)	145 (29.0)	<0.0001
Age group, *n* (%)					
18–29	1399 (25.4)	279 (20.0)	631 (45.3)	483 (34.7)	<0.0001
30–39	1104 (20.1)	288 (26.2)	529 (48.1)	283 (25.7)	0.01
40–49	905 (16.4)	299 (33.1)	384 (42.5)	221 (24.4)	(ref)
50–59	864 (15.7)	327 (38.0)	364 (42.2)	171 (19.8)	0.008
60–69	794 (14.4)	393 (49.5)	293 (36.9)	108 (13.6)	<0.0001
≥70	434 (7.9)	237 (54.5)	161 (37.2)	36 (8.3)	<0.0001
Gender, *n* (%)					
Man	2588 (47.1)	1000 (38.8)	1047 (40.6)	533 (20.7)	(ref)
Woman	2771 (50.5)	801 (29.0)	1265 (45.7)	702 (25.4)	<0.0001
Nonbinary^c^ or transgender	133 (2.4)	15 (11.0)	50 (37.9)	67 (51.0)	<0.0001
Health insurance, *n* (%)					
Any private insurance	2384 (43.6)	860 (36.1)	1043 (43.9)	476 (20.0)	(ref)
Public insurance only	1953 (35.7)	615 (31.6)	833 (42.8)	499 (25.6)	<0.0001
Uninsured	1137 (20.8)	337 (29.7)	475 (41.8)	324 (28.5)	<0.0001
Physical health, *n* (%)					
Excellent/very good/good	4015 (73.0)	1518 (37.9)	1732 (43.2)	757 (18.9)	(ref)
Fair/poor	1485 (27.0)	306 (20.6)	631 (42.6)	545 (36.8)	<0.0001
Chronic conditions, *n* (%)					
One or more	1694 (30.8)	485 (28.7)	706 (41.8)	498 (29.5)	<0.0001
None	3804 (69.2)	1336 (35.2)	1656 (43.6)	804 (21.2)	(ref)
Immigration status, *n* (%)					
US-born citizen	4276 (77.8)	1366 (32.0)	1816 (42.6)	1083 (25.4)	(ref)
Foreign-born citizen/legal resident	946 (17.2)	350 (37.0)	410 (43.4)	185 (19.6)	<0.0001
Undocumented	275 (5.0)	108 (39.2)	135 (49.3)	32 (11.6)	<0.0001
Education, *n* (%)					
Less than high school graduate	498 (9.1)	162 (32.7)	196 (39.5)	138 (27.8)	0.01
High school/GED	1791 (32.6)	651 (36.4)	718 (40.1)	421 (23.5)	0.52
Some college/vocational school	1690 (30.7)	507 (30.1)	720 (42.7)	460 (27.3)	<0.0001
College graduate or more	1520 (27.6)	504 (33.2)	730 (48.1)	284 (18.7)	(ref)
Marital status, *n* (%)					
Married/domestic partnership	2551 (46.4)	951 (37.4)	1090 (42.9)	503 (19.8)	<0.0001
Not married	2949 (53.6)	873 (29.6)	1272 (43.2)	799 (27.1)	(ref)
Family annual income,^d^ *n* (%)					
<$20,000	1095 (22.8)	332 (30.6)	409 (37.6)	346 (31.9)	<0.0001
$20,000–$59,000	1921 (40.0)	583 (30.3)	846 (44.0)	492 (25.6)	<0.0001
$60,000–$99,000	974 (20.3)	321 (33.1)	446 (45.9)	203 (20.9)	<0.0001
≥$100,000	818 (17.0)	339 (41.4)	343 (42.0)	136 (16.6)	(ref)

^a^ Anxiety-depression was measured with the PHQ-4 and scored as none (0), very mild (1–2), mild (3–5), moderate (6–8), or severe (9–12) and then collapsed to none, very mild/mild, or moderate/severe, due to small numbers in some of the categories.

^b^ Cochran–Armitage trend tests were used to compare anxiety-depression symptom severity across demographics; *p*-value <0.05 was considered statistically significant.

^c^ Nonbinary includes individuals who reported being nonbinary, gender fluid, gender queer, “other,” and no gender.

^d^ Collected by YouGov at enrollment into panel and updated every 6 months.

GED, general educational development; PHQ-4, Patient Health Questionnaire-4.

Among the Asian subpopulations, Filipino (23.1%) and Asian Indian (21.2%) adults had the highest prevalence of moderate/severe anxiety-depression followed by other Asian adults (20.7); Chinese (13.5%) and Japanese (12.3%) adults had a lower prevalence compared with the other Asian groups. The prevalence of moderate/severe anxiety-depression was similar among Native Hawaiian (26.8%) and Pacific Islander (28.7%) adults.

There was a stepwise inverse association between age and moderate/severe anxiety-depression, with the highest prevalence among those ages 18–29 years (34.7%) and the lowest for adults ≥70 years (8.3%). Nonbinary or transgender adults (51.0%) had twice the prevalence of either men (20.7%) or women (25.4%). There was also a stepwise inverse association between family income and moderate/severe anxiety-depression, with the highest prevalence among those reporting <$20,000 (31.9%) and the lowest for income ≥$100,000 (16.6%).

After adjustment, compared with white adults, moderate/severe anxiety-depression symptoms were significantly less prevalent among Asian (adjusted odds ratio [aOR]=0.44, 95% confidence interval [CI]=0.34–0.58), black (aOR=0.49, 95% CI=0.38–0.63), English-speaking Latino (aOR=0.62, 95% CI=0.45–0.85), Spanish-speaking Latino (aOR=0.31, 95% CI=0.22–0.44), and Native Hawaiian/Pacific Islander (aOR=0.66, 95% CI=0.49–0.90) adults (Table 2).

**Table 2.** Adjusted Associations Between Race-Ethnicity, Anxiety-Depression Symptoms, Perceived Stress, and Loneliness

	Anxiety-depression*^a^*		*Perceived stress^b^*		*Loneliness^c^*	
	*Very mild/mild aOR (95% CI)^d^*	*Moderate/severe aOR (95% CI)^d^*	*Mild aOR (95% CI)^d^*	*Moderate/severe aOR (95% CI)^d^*	*Almost never/sometimes aOR (95% CI)^d^*	*Fairly often/very often aOR (95% CI)^d^*
Race-ethnicity						
American Indian/Alaska Native	1.12 (0.85–1.48)	1.07 (0.78–1.46)	0.84 (0.63–1.11)	0.92 (0.70–1.22)	0.99 (0.77–1.29)	0.97 (0.72–1.31)
Asian	0.76 (0.62–0.95)	0.44 (0.34–0.58)	0.73 (0.59–0.92)	0.58 (0.46–0.74)	0.84 (0.68–1.03)	0.52 (0.40–0.67)
Black/African American	0.64 (0.51–0.79)	0.49 (0.38–0.63)	0.57 (0.45–0.71)	0.56 (0.44–0.70)	0.64 (0.52–0.79)	0.51 (0.39–0.65)
Latino						
English speaking	0.92 (0.71–1.20)	0.62 (0.45–0.85)	0.68 (0.51–0.89)	0.58 (0.44–0.76)	0.73 (0.57–0.94)	0.50 (0.37–0.68)
Spanish speaking	0.81 (0.62–1.05)	0.31 (0.22–0.44)	0.74 (0.56–0.98)	0.41 (0.30–0.54)	0.75 (0.58–0.96)	0.33 (0.23–0.46)
Native Hawaiian/Pacific Islander	0.77 (0.59–1.00)	0.66 (0.49–0.90)	0.72 (0.54–0.96)	0.74 (0.56–0.97)	0.76 (0.59–0.99)	0.65 (0.48–0.88)
White	1.00 (ref)	1.00 (ref)	1.00 (ref)	1.00 (ref)	1.00 (ref)	1.00 (ref)
Multiracial	1.63 (1.22–2.17)	1.07 (0.76–1.49)	1.00 (0.75–1.34)	1.04 (0.78–1.39)	1.08 (0.82–1.41)	1.21 (0.90–1.63)

^a^ Anxiety-depression was assessed with the PHQ-4; scoring=none (0; reference), very mild/mild (1–5), or moderate/severe (6–12).

^b^ Perceived stress was assessed with a six-item adapted version of the PSS-10; scoring=low (1; reference), mild (1.1–2), or moderate/severe stress (2.1–5).

^c^ Loneliness was assessed with a single item that asks how often in the past month they felt lonely and isolated; scoring=never (1; reference), almost never/sometimes (2–3), or fairly often/very often (4–5).

^d^ All results are adjusted for age group, gender (male, female, nonbinary/transgender), education, and self-reported physical health (poor/not poor).

aOR, adjusted odds ratio; CI, confidence interval; PSS-10, Perceived Stress Scale-10.

### Perceived stress

One-third of the sample (34.3%, *n*=1887) reported moderate/severe stress (Table 3). Compared with white adults (moderate/severe=33.7%), American Indian/Alaska Native (40.9%) and multiracial adults (44.6%) were more likely, and Spanish-speaking Latino (25.4%) adults less likely to report moderate/severe stress (all *p*<0.05). No statistically significant differences were seen between white and Asian (30.3%), black (33.4%), English-speaking Latino (33.2%), and Native Hawaiian/Pacific Islander (38.6%) adults. Among the Latino subpopulations, Puerto Rican (38.4%) and Mexican (29.9%) adults had the highest prevalence of moderate/severe stress. Among the Asian subpopulations, Filipino (37.5%), other Asian (35.1%), and Asian Indian (33.2%) adults had the highest prevalence of moderate/severe stress. The prevalence of moderate/severe perceived stress was similar among Native Hawaiian (38.8%) and Pacific Islander (38.4%) adults.

**Table 3.** Participant Characteristics, Weighted to Be Nationally Representative Within Racial-Ethnic Groups, Stratified by Perceived Stress

	*Overall*	*Perceived stress^a^*			
		*Low*	*Mild*	*Moderate/severe*	****p*** ^b^*
Total, *n*	5500	2066 (37.6)	1547 (28.1)	1887 (34.3)	—
Race-ethnicity, *n* (%)					
American Indian/Alaska Native	500 (9.1)	164 (32.7)	132 (26.4)	205 (40.9)	0.03
Asian	1000 (18.2)	392 (39.2)	305 (30.5)	303 (30.3)	0.07
Asian Indian	175 (3.2)	44 (25.4)	72 (41.3)	58 (33.2)	—
Chinese	274 (5.0)	121 (44.2)	93 (33.9)	60 (21.9)	—
Filipino	161 (2.9)	64 (39.7)	37 (22.9)	60 (37.5)	—
Japanese	132 (2.4)	54 (40.5)	44 (33.5)	34 (26.0)	—
Other Asian	258 (4.7)	109 (42.2)	58 (22.6)	91 (35.1)	—
Black/African American	1000 (18.2)	428 (42.8)	238 (23.8)	334 (33.4)	0.06
Latino	1000 (18.2)	416 (41.6)	292 (29.2)	293 (29.3)	0.006
*English speaking*	496 (49.6)	198 (39.9)	134 (26.9)	165 (33.2)	0.32
*Spanish speaking*	504 (50.4)	218 (43.3)	158 (31.4)	128 (25.4)	<0.0001
Mexican/Mexican American/Chicano	528 (9.6)	221 (41.9)	149 (28.3)	158 (29.9)	—
Puerto Rican	115 (2.1)	40 (34.6)	31 (27.0)	44 (38.4)	—
Cuban/Dominican Republic	85 (1.5)	34 (39.7)	27 (32.2)	24 (28.2)	—
Central American	80 (1.4)	40 (50.7)	19 (23.7)	20 (25.6)	—
South American	99 (1.8)	38 (38.6)	37 (37.7)	23 (23.7)	—
Other Hispanic/Latino	94 (1.7)	43 (45.8)	28 (29.8)	23 (24.4)	—
Native Hawaiian/Pacific Islander	500 (9.1)	178 (35.7)	129 (25.8)	193 (38.6)	0.28
Native Hawaiian	274 (5.0)	104 (38.0)	63 (23.2)	106 (38.8)	—
Pacific Islander	226 (4.1)	74 (32.8)	65 (28.9)	87 (38.4)	—
White	1000 (18.2)	357 (35.7)	306 (30.6)	337 (33.7)	(ref)
Multiracial	500 (9.1)	131 (26.3)	146 (29.1)	223 (44.6)	<0.0001
Age group, *n* (%)					
18–29	1399 (25.4)	358 (25.6)	387 (27.7)	654 (46.7)	<0.0001
30–39	1104 (20.1)	338 (30.6)	343 (31.1)	423 (38.3)	0.338
40–49	905 (16.4)	309 (34.2)	249 (27.6)	346 (38.3)	(ref)
50–59	864 (15.7)	366 (42.4)	223 (25.8)	275 (31.8)	<0.0001
60–69	794 (14.4)	438 (55.2)	213 (26.8)	143 (18.0)	<0.0001
≥70	434 (7.9)	256 (59.1)	132 (30.4)	46 (10.6)	<0.0001
Gender, *n* (%)					
Man	2588 (47.1)	1121 (43.3)	716 (27.7)	750 (29.0)	(ref)
Woman	2771 (50.5)	917 (33.1)	798 (28.8)	1055 (38.1)	<0.0001
Nonbinary^c^ or transgender	133 (2.4)	20 (15.1)	32 (23.7)	81 (61.2)	<0.0001
Health insurance, *n* (%)					
Any private insurance	2384 (43.6)	954 (40.0)	720 (30.2)	711 (29.8)	(ref)
Public insurance only	1953 (35.7)	726 (37.2)	513 (26.3)	714 (36.6)	<0.0001
Uninsured	1137 (20.8)	377 (33.2)	308 (27.1)	451 (39.7)	<0.0001
Physical health, *n* (%)					
Excellent/very good/good	4015 (73.0)	1,713 (42.7)	1,132 (28.2)	1,171 (29.2)	(ref)
Fair/poor	1,485 (27.0)	353 (23.8)	415 (28.0)	716 (48.3)	<0.0001
Chronic conditions, *n* (%)					
One or more	1,694 (30.8)	559 (33.0)	482 (28.5)	653 (38.6)	<0.0001
None	3,804 (69.2)	1,505 (39.6)	1,065 (28.0)	1,234 (32.4)	(ref)
Immigration status, *n* (%)					
US-born citizen	4,276 (77.8)	1,593 (37.3)	1,154 (27.0)	1,529 (35.8)	(ref)
Foreign-born citizen/legal resident	946 (17.2)	370 (39.1)	293 (31.0)	283 (29.9)	0.012
Undocumented	275 (5.0)	104 (37.8)	99 (36.0)	72 (26.3)	0.059
Education, *n* (%)					
Less than high school graduate	498 (9.1)	179 (35.9)	142 (28.6)	177 (35.5)	0.12
High school/GED	1,791 (32.6)	774 (43.2)	438 (24.5)	579 (32.3)	0.19
Some college/vocational school	1,690 (30.7)	548 (32.4)	469 (27.7)	673 (39.8)	<0.0001
College graduate or more	1,520 (27.6)	565 (37.2)	498 (32.7)	458 (30.1)	(ref)
Marital status, *n* (%)					
Married/domestic partnership	2,551 (46.4)	1,065 (41.7)	727 (28.5)	759 (29.7)	<0.0001
Not married	2,949 (53.6)	1,001 (34.0)	819 (27.8)	1,128 (38.3)	(ref)
Family annual income,^d^ *n* (%)					
<$20,000	1095 (22.8)	350 (32.0)	256 (23.4)	489 (44.7)	<0.0001
$20,000–$59,000	1921 (40.0)	674 (35.1)	573 (29.8)	674 (35.1)	<0.0001
$60,000–$99,000	974 (20.3)	373 (38.3)	298 (30.6)	303 (31.1)	<0.0001
≥$100,000	818 (17.0)	381 (46.6)	230 (28.1)	207 (25.3)	

^a^ Perceived stress was measured by a six-item adapted version of the PSS-10 and scored as low (1), mild (1.1–2), moderate (2.1–3), or severe (4–5) and then collapsed to low, mild, or moderate/severe stress due to small numbers in some of the categories.

^b^ Cochran–Armitage trend tests were used to compare perceived stress across demographics; *p*-value <0.05 was considered statistically significant.

^c^ Nonbinary includes individuals who reported being nonbinary, gender fluid, gender queer, “other,” and no gender.

^d^ Collected by YouGov at enrollment into panel and updated every 6 months.

Similar to anxiety-depression, there was a stepwise inverse association between age and moderate/severe stress (except age groups of 30–39 and 40–49 years were the same), with the highest prevalence among those 18–29 years (46.7%) and the lowest among adults ≥70 years (10.6%). Nonbinary or transgender adults (61.2%) had twice the prevalence of moderate/severe perceived stress compared with men (29.0%) or women (38.1%). There was a stepwise inverse association between family income and moderate/severe stress, with the highest prevalence among those reporting <$20,000 (44.7%) and the lowest for those reporting ≥$100,000 (25.3%).

In adjusted analyses, compared with white adults, moderate/severe stress was significantly less prevalent among Asian (aOR=0.58, 95% CI=0.46–0.74), black (aOR=0.56, 95% CI=0.44–0.70), English-speaking Latino (aOR=0.58, 95% CI=0.44–0.76), Spanish-speaking Latino (aOR=0.41, 95% CI=0.30–0.54), and Native Hawaiian/Pacific Islander (aOR=0.74, 95% CI=0.56–0.97) adults (Table 2).

### Loneliness-isolation

About 21% reported feeling lonely-isolated fairly/very often (Table 4). Compared with white adults (fairly often/very often=23.2%), multiracial adults (33.2%) were more likely, and Asian (16.9%), black (19.6%), English-speaking Latino (19.2%), and Spanish-speaking Latino (12.9%) adults less likely to report feeling lonely-isolated fairly/very often (all *p*<0.05). Loneliness-isolation was more common among English-speaking Latino adults, compared with Spanish-speaking Latino adults, *p*=0.02. Among the Latino subpopulations, Puerto Rican adults had the highest prevalence of feeling lonely-isolated fairly/very often (25.7%). Among the Asian subpopulations, Asian Indian (24.2%) adults had the highest prevalence of feeling lonely-isolated fairly/very often. The prevalence of feeling lonely-isolated fairly/very often was similar among Native Hawaiian (22.7%) and Pacific Islander (23.4%) adults.

**Table 4.** Participant Characteristics, Weighted to Be Nationally Representative Within Racial-Ethnic Groups, Stratified by Loneliness-Isolation

	*Overall*	*Loneliness-isolation^a^*			
		*Never*	*Almost never/sometimes*	*Fairly often/very often*	****p*** ^b^*
Total, *n*	5492	2135 (38.9)	2185 (39.8)	1172 (21.3)	—
Race-ethnicity, *n* (%)					
American Indian/Alaska Native	500 (9.1)	162 (32.5)	200 (40.2)	136 (27.3)	0.05
Asian	1000 (18.2)	399 (39.9)	432 (43.2)	169 (16.9)	0.003
Asian Indian	175 (3.2)	52 (29.5)	81 (46.3)	42 (24.2)	—
Chinese	274 (5.0)	121 (44.2)	111 (40.5)	42 (15.4)	—
Filipino	161 (2.9)	67 (41.9)	71 (44.2)	22 (14.0)	—
Japanese	132 (2.4)	53 (40.4)	63 (47.7)	16 (11.9)	—
Other Asian	258 (4.7)	106 (41.1)	106 (41.0)	46 (18.0)	—
Black/African American	1000 (18.2)	443 (44.3)	361 (36.1)	196 (19.6)	0.001
Latino	1000 (18.2)	439 (44.0)	399 (40.0)	159 (16.0)	<0.0001
*English speaking*	496 (49.6)	206 (41.7)	194 (39.1)	95 (19.2)	0.03
*Spanish speaking*	504 (50.4)	232 (46.3)	205 (40.9)	65 (12.9)	<0.0001
Mexican/Mexican American/Chicano	528 (9.6)	237 (45.1)	207 (39.5)	81 (15.4)	—
Puerto Rican	115 (2.1)	43 (37.6)	42 (36.7)	29 (25.7)	—
Cuban/Dominican Republic	85 (1.5)	35 (40.9)	32 (38.2)	18 (20.9)	—
Central American	80 (1.4)	38 (47.7)	33 (41.4)	9 (10.9)	—
South American	99 (1.8)	45 (45.0)	46 (46.3)	9 (8.7)	—
Other Hispanic/Latino	94 (1.7)	42 (44.3)	38 (40.9)	14 (14.9)	—
Native Hawaiian/Pacific Islander	500 (9.1)	193 (38.7)	191 (38.3)	114 (23.0)	0.57
Native Hawaiian	274 (5.0)	104 (37.9)	108 (39.5)	62 (22.7)	—
Pacific Islander	226 (4.1)	89 (39.7)	83 (36.9)	52 (23.4)	—
White	1000 (18.2)	365 (36.5)	403 (40.3)	232 (23.2)	(ref)
Multiracial	500 (9.1)	134 (26.9)	200 (39.9)	166 (33.2)	<0.0001
Age group, *n* (%)					
18–29	1399 (25.4)	390 (27.9)	558 (39.9)	451 (32.2)	<0.0001
30–39	1104 (20.1)	382 (34.7)	488 (44.3)	232 (21.1)	0.31
40–49	905 (16.4)	349 (38.6)	358 (39.7)	195 (21.6)	(ref)
50–59	864 (15.7)	380 (44.0)	337 (39.0)	146 (17.0)	0.005
60–69	794 (14.4)	399 (50.3)	286 (36.1)	108 (13.6)	<0.0001
≥70	434 (7.9)	236 (54.4)	157 (36.2)	41 (9.4)	<0.0001
Gender, *n* (%)					
Man	2588 (47.1)	1110 (43.0)	991 (38.4)	482 (18.7)	(ref)
Woman	2771 (50.5)	994 (35.9)	1142 (41.3)	632 (22.8)	<0.0001
Nonbinary^c^ or transgender	133 (2.4)	24 (18.3)	49 (37.4)	59 (44.2)	<0.0001
Health insurance, *n* (%)					
Any private insurance	2384 (43.6)	968 (40.6)	972 (40.8)	443 (18.6)	(ref)
Public insurance only	1953 (35.7)	755 (38.8)	735 (37.7)	457 (23.5)	0.003
Uninsured	1137 (20.8)	401 (35.3)	466 (41.1)	268 (23.6)	<0.0001
Physical health, *n* (%)					
Excellent/very good/good	4015 (73.0)	1731 (43.2)	1549 (38.6)	731 (18.2)	(ref)
Fair/poor	1485 (27.0)	404 (27.3)	635 (42.9)	442 (29.8)	<0.0001
Chronic conditions, *n* (%)					
One or more	1694 (30.8)	567 (33.6)	724 (42.9)	398 (23.6)	<0.0001
None	3804 (69.2)	1566 (41.2)	1460 (38.4)	774 (20.4)	(ref)
Immigration status, *n* (%)					
US-born citizen	4276 (77.8)	1613 (37.8)	1672 (39.2)	984 (23.1)	(ref)
Foreign-born citizen/legal resident	946 (17.2)	397 (42.0)	386 (40.9)	162 (17.1)	<0.0001
Undocumented	275 (5.0)	125 (45.6)	124 (45.3)	25 (9.1)	<0.0001
Education, *n* (%)					
Less than high school graduate	498 (9.1)	206 (41.6)	178 (35.9)	112 (22.5)	0.86
High school/GED	1791 (32.6)	765 (42.8)	664 (37.2)	359 (20.1)	0.25
Some college/vocational school	1690 (30.7)	579 (34.3)	693 (41.0)	416 (24.6)	<0.0001
College graduate or more	1520 (27.6)	585 (38.5)	650 (42.7)	286 (18.8)	(ref)
Marital status, *n* (%)					
Married/domestic partnership	2551 (46.4)	1182 (46.4)	998 (39.2)	367 (14.4)	<0.0001
Not married	2949 (53.6)	953 (32.4)	1187 (40.3)	805 (27.3)	(ref)
Family annual income,^d^ *n* (%)					
<$20,000	1095 (22.8)	372 (34.0)	415 (37.9)	308 (28.1)	<0.0001
$20,000–$59,000	1921 (40.0)	692 (36.2)	822 (43.0)	400 (20.9)	<0.0001
$60,000–$99,000	974 (20.3)	371 (38.1)	407 (41.8)	196 (20.1)	<0.0001
≥$100,000	818 (17.0)	390 (47.7)	309 (37.7)	119 (14.6)	(ref)

^a^ Loneliness-isolation was measured with a single item that asks how often in the past month they felt lonely and isolated, with responses and scoring of never (1), almost never (2), sometimes (3), fairly often (4), or very often (5), and then collapsed to never, almost never, sometimes, or fairly often/very often, due to small numbers in the higher categories.

^b^ Cochran–Armitage trend tests were used to compare loneliness-isolation across demographics; *p*-value <0.05 was considered statistically significant.

^c^ Nonbinary includes individuals who reported being nonbinary, gender fluid, gender queer, “other,” and no gender.

^d^ Collected by YouGov at enrollment into panel and updated every 6 months.

There was a stepwise inverse association between age and feeling lonely-isolated fairly/very often (except age groups of 30–39 and 40–49 years were the same); the highest prevalence was among those ages 18–29 years (32.2%) and the lowest for adults ≥70 years (9.4%). Nonbinary or transgender adults (44.2%) had more than twice the prevalence of feeling lonely-isolated fairly/very often compared with men (18.7%) or women (22.8%). There was a stepwise inverse association between family income and feeling lonely-isolated fairly/very often, with the highest prevalence among those reporting <$20,000 (28.1%) and the lowest for those reporting ≥$100,000 (14.6%).

In adjusted analyses, compared with white adults, feeling lonely-isolated fairly/very often was significantly less prevalent among Asian (aOR=0.52, 95% CI=0.40–0.67), black (aOR=0.51, 95% CI=0.39–0.65), English-speaking Latino (aOR=0.50, 95% CI=0.37–0.68), Spanish-speaking Latino (aOR=0.33, 95% CI=0.23–0.46), and Native Hawaiian/Pacific Islander (aOR=0.65, 95% CI=0.48–0.88) adults (Table 2).

### Open-ended item

There were 5442 responses to the open-ended question; 767 (14.1%) were related to psychological distress, and 11 themes emerged. The most prevalent was *disrupted lives and routines* (64.1% of responses). Disruptions reported included routines related to grocery shopping, religious practices, social gatherings, dating, weddings, travel, work, and school. *Feeling stressed* was next most common (12.6%), including generalized stress and stress specific to work, finances, family relationships, and media/news consumption. *Worsening of personal relationships* (10.0%) was next most common. *Anxiety and worry* were mentioned in 8.7% of responses (generalized anxiety, worrying about the future and others' well-being, fear of the virus, and being in close proximity to others). *Feeling isolated and lonely* were concerns in 8.5% of responses (e.g., inability to see friends and family). Depressive symptoms were mentioned in 6% of responses (feelings of hopelessness, prolonged sadness).

## Discussion

This study examined racial-ethnic differences in experiences of anxiety-depressive symptoms, perceived stress, and loneliness-isolation among nationally representative samples of the major US racial-ethnic populations. Overall, 23.7% reported moderate/severe anxiety-depression, 34.3% reported moderate/severe perceived stress, and 21.4% reported loneliness-isolation fairly/very often. On all three measures, American Indian/Alaska Native and multiracial adults reported the highest levels in unadjusted analyses. Multiracial adults had the highest prevalence of moderate/severe perceived stress (44.6%) and fairly/very often feeling lonely-isolated (33.3%); American Indian/Alaska Native adults had the highest levels of moderate/severe anxiety-depression (32.1%). Although differences between each of these groups and white adults were attenuated after adjustment (for age, gender, education, and self-rated physical health), the prevalence of psychological distress and disproportionate burden among American/Indian/Alaska Native and multiracial adults are notable.

Our distress prevalence estimates are similar to prior studies.^3,28^ A longitudinal comparison of waves of the US Census Bureau Household Pulse Survey conducted between August 2020 and February 2021 found that anxiety and depressive symptoms among US adults peaked during December 9–21, 2020, and January 8–18, 2021, survey waves.^29^ This period overlaps with our survey period, and thus, our nuanced results by racial-ethnic group may reflect peak pandemic-related levels.

Higher psychological distress in American Indian/Alaska Native adults may be explained, in part, by lower income, wealth, and power persisting since colonization, compared with white adults.^30^ Historical and childhood trauma, forced geographical isolation and segregation, limited economic opportunities, and racism have been major drivers of health disparities among American Indian/Alaska Native populations.^31^ They are more likely than white adults to experience poor health, disability, and comorbidities, and have not experienced improvements in life expectancy seen in other US racial-ethnic groups.^32^

Limited health research has focused on multiracial people, despite being the fastest-growing racial group in the United States.^33^ The impact of structural racism and intersectionality of multiple marginalized identities on this group's health is poorly understood.^34^ Another nationally representative study found that multiracial respondents had the highest anxiety-depression symptoms compared with most other racial-ethnic groups.^35^ Behavioral and phenotypic invalidation (e.g., being treated differently because others perceive one does not behave/look similar to someone from a racial-ethnic group with which one identifies) and family discrimination (e.g., family member says something negative about multiracial people) are unique forms of discrimination faced by multiracial persons.^33^ Both invalidation types are associated with anxiety-depressive symptoms and stress.^33^

We found consistency in our results across all three psychological distress measures. Asian, black/African American, Latino (English and Spanish speaking), and Native Hawaiian/Pacific Islander adults were less likely than white adults to report severe anxiety-depression, perceived stress, and loneliness-isolation. Our findings contradict studies that found symptoms of psychological distress to be more common among Latino and other adults of color^6–11^ than in white adults.^8,10^ The resilience of racial-ethnic minorities during the pandemic may reflect strength that is rooted in collective, historical hardships and lessons learned.^36^

Such lessons may include identification of effective methods for utilizing social networks, civic engagement, and shared values for the dissemination of health information and community resources to mitigate harmful consequences in times of crises. For example, the higher COVID-19 vaccination rates among tribal communities relative to other racial-ethnic groups were attributed to mobilization of community resources, decentralized tribal control of information, and integration of cultural values in vaccination campaigns.^37^ Perhaps perceiving racism and social and health inequalities as a collective or common fate that is tied to one's ethnic identity results in normalization of social disadvantage, community solidarity, and advocacy that buffer against the negative impact of COVID-19.^38^

Psychological resiliency has been defined as an individual's internalized ability to adapt effectively to stressors and adversities.^39^ Studies conducted among Latino, black, and Asian American adults and adolescents suggest that resiliency among the minoritized groups in the United States operates through a variety of mechanisms, including high levels of self-esteem, cultural connectedness/identity, mastery, familial and other social support, and spirituality or religious involvement.^39–42^ Relative to white adults, African American and Latino adults have reported higher levels of optimism and life satisfaction, and lower levels of stress, which have been attributed to community and religious factors and determination against adversity.^43^ Alternatively, higher levels of stigma associated with psychological distress among racially and ethnically minoritized groups may have resulted in underreporting of the symptom burden using the self-report measures used in our study.

Finally, we must consider also that our cross-sectional findings may not capture the effects of unrecognized internalized stress resulting from the pandemic and other chronic traumas that can cause significant deleterious long-term effects to the mental and physical health of US minority populations.

Because we stratified Latino adults by language preference and subpopulation, these results merit attention. English-speaking Latino adults did worse than Spanish-speaking Latino adults, as did Puerto Rican individuals, compared with other Latino groups. Spanish-speaking Latino individuals report greater stigma related to mental health than white individuals,^44^ or may underreport psychological symptoms because they are not captured using conventional measures (e.g., illness conceptualizations may differ or distress may manifest as somatic symptoms).^45,46^ Our findings that Puerto Rican adults experience more distress are consistent with prior results.^47^ The higher risk of psychological distress among Puerto Rican adults relative to some of the other Latino subpopulations could be due to a higher risk of socioeconomic disparities associated with colonialism by the United States^48^ or loss of social support and greater exposure to racial discrimination once they relocate from the island to the mainland.^49^

Although Asian adults overall reported less distress than white adults, there were differences among subpopulations. Filipino, Asian Indian, and other Asian subpopulations did worse compared with the Chinese and Japanese adults. A California population-based study found the highest prevalence of psychological distress in Korean adults; Chinese, Filipino, Vietnamese, and Japanese adults reported lower levels of distress than white adults.^50^ In our study, Koreans adults made up 30% (and the largest subgroup) of the group classified as “other Asian”; therefore, if the national prevalence among Koreans relative to the other Asian subpopulations is consistent with the results of the California study, our results for the other Asian group may reflect largely the experiences of Koreans.

Based on our findings and the literature, future research needs to focus on identifying potential mechanisms of resiliency and adaptive coping that can be supported among vulnerable population groups so that they can withstand the negative consequences of pandemics, natural disasters, and chronic diseases, which disproportionately affect them. We need to better understand why some racial-ethnic minority groups tend to report better psychological well-being despite greater socioeconomic deprivation and structural racism.

Research that identifies ameliorable risk and resilience factors among racial-ethnic minority individuals who reported higher levels of distress in our study, that is, American Indian/Alaska Native and multiracial adults, is also critical. Finally, more granular research on how mechanisms of poor and better psychological distress outcomes differ by subpopulations and their characteristics, for example, language, immigrant status, historical experiences, socioeconomic and other structural factors, could help to increase the effectiveness of mental health interventions.

The limitations of our study include using online panel recruitment, which likely negatively affected our ability to recruit vulnerable persons with limited access to internet resources. Although the measures of depression/anxiety and stress used in our study have been widely used in diverse populations, because of the unexpected findings, further research could explore the extent to which our observed group differences could reflect cultural biases.^51^ Bias can be introduced through culturally mediated differences in perceptions of the meaning of items, or in the cognitive processes of responding to items. We administered the survey in English and Spanish, so persons who prefer other languages were not included. Also, individuals who lacked proficiency in English could have completed the survey in English.

Finally, we did not collect information on the heritage (national origin) of white or black individuals in our study, which could mask heterogeneity within these groups on the outcomes of interest.

## Conclusions

Psychological distress levels varied by race-ethnicity; American Indian/Alaska Native and multiracial adults were at higher risk than white adults. Despite their generally worse socioeconomic profile and histories of structural racism, black/African American, Latino, and Native Hawaiian/Pacific Islander adults did better than white adults. Screening to assess the pandemic's longer term psychological sequelae, especially among high-risk populations experiencing chronic stress and limited access to mental health resources, is necessary. Data by race, ethnicity, and language can be applied to effectively tailor efforts to mitigate COVID-19's impact on the psychological well-being of the US population, while being responsive to the specific needs of various population groups.

## Abbreviations Used

aOR: adjusted odds ratio
CI: confidence interval
CURB: COVID-19's Unequal Racial Burden
GED: general educational development
PHQ-4: Patient Health Questionnaire-4
PSS-10: Perceived Stress Scale-10
